# Sorption Kinetics and Sequential Adsorption Analysis of Volatile Organic Compounds on Mesoporous Silica

**DOI:** 10.1021/acsomega.2c05608

**Published:** 2022-11-16

**Authors:** Pallabi Samaddar, Jinchuang Hu, Nirmalay Barua, Yixian Wang, Tse-Ang Lee, Maša Prodanović, Zoya Heidari, Tanya Hutter

**Affiliations:** † Walker Department of Mechanical Engineering, The University of Texas at Austin, Austin, Texas78712, United States; ‡ Hildebrand Department of Petroleum and Geosystems Engineering, The University of Texas at Austin, Austin, Texas78712, United States; § Materials Science and Engineering Program and Texas Materials Institute, The University of Texas at Austin, Austin, Texas78712, United States; ∥ Center for Subsurface Energy and the Environment, The University of Texas at Austin, Austin, Texas78712, United States

## Abstract

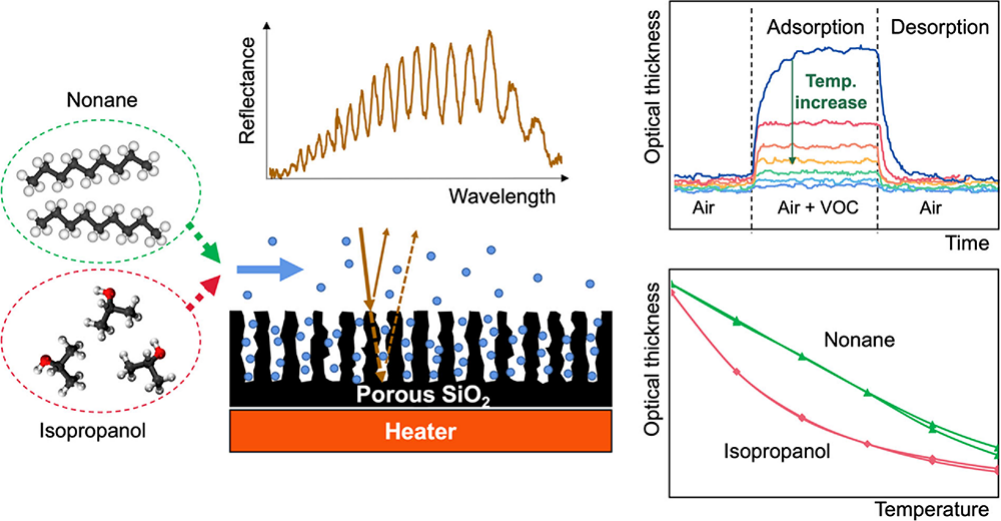

Adsorption–desorption behaviors of polar and nonpolar volatile organic compounds (VOCs), namely, isopropanol and nonane, on mesoporous silica were studied using optical reflectance spectroscopy. Mesoporous silica was fabricated via electrochemical etching of silicon and subsequent thermal oxidation, resulting in an average pore diameter of 11 nm and a surface area of approximately 493 m^2^/g. The optical thickness of the porous layer, which is proportional to the number of adsorbed molecules, was measured using visible light reflectance interferometry. In situ adsorption and desorption kinetics were obtained for various mesoporous silica temperatures ranging from 10 to 70 °C. Sorption as a function of temperature was acquired for isopropanol and nonane. Sequential adsorption measurements of isopropanol and nonane were performed and showed that, when one VOC is introduced immediately following another, the second VOC displaces the first one regardless of the VOC’s polarity and the strength of its interaction with the silica surface.

## 1 Introduction

Adsorption of molecules on high-surface-area materials is a fundamental process critical to many fields of basic and applied chemical research. Properties of adsorption, desorption, and interactions in nanoporous materials (i.e., those with pores smaller than 100 nm) are significantly different from interactions on flat surfaces. They are highly dependent on the nanofeature sizes, connectivity, surface shape, and chemistry and have a wide range of applications in material science.^1,2^ The same processes are also studied when characterizing subsurface rocks, such as mudrocks, which can contain nanoscale pores.

Sorption hysteresis is a phenomenon where adsorption and desorption isotherms do not overlap, and it commonly occurs in porous materials.^1^ In small pores, the adsorbed layers of gas on solid surfaces densify faster and can turn into liquid at a lower pressure than on a flat surface. This phenomenon is called capillary condensation. Capillary evaporation usually happens at a comparatively lower pressure than condensation; thus, hysteresis in sorption and desorption curves occurs. Factors such as the temperature, pore diameter, pore shape, surface tension, molecular chain length, and wettability^2−7^ as well as the interconnectivity of the nanoporous network where applicable^8^ affect the shape of the hysteresis curve.

Mesoporous silicas have been utilized for the study of multiple aspects of capillary condensation–evaporation or adsorption–desorption hysteresis. The interest in these materials rises from their structural characteristics including high pore volumes, large surface areas, uniform pore sizes, and thermal stability.^9,10^ A myriad of mesoporous silica materials, such as MCM-48 and KIT-6, which have a 3D bicontinuous cubic pore structure,^11−13^ and SBA-15 and MCM-41, which have a hexagonal arrangement of uniform mesopores,^14−16^ have been used to study the mechanism of adsorption–desorption hysteresis associated with the capillary condensation. Previous studies of cage-like ordered silica, cylindrical pores, and mesoporous molecular sieves like MCM-41 normally rely on nitrogen/argon adsorption–desorption hysteresis at cryogenic temperatures.^17,18^ The sorption affinity of various VOCs on the surface of silica has been explained in terms of polarizability of the adsorbed organic compounds on mesoporous silicate materials (e.g., MCM-48 and MCM-41).^19−22^ In general, silicate materials exhibit more affinity toward the polar VOCs in comparison with non-polar VOCs.

Understanding adsorption of VOCs can help with designing materials for separation, and a recent study showed the potential of silica as a separation material for VOC mixtures using dynamic adsorption.^21,23−25^ Moreover, most studies provide the adsorption capacity for VOCs but not the kinetics.^26,27^ Understanding the temperature-dependent surface dynamics of nanoporous silica materials requires further study. The literature also lacks information about the equilibrium and sorption kinetics at different temperatures, which is expected to be adsorbate/adsorbent-dependent, as well as studies of sequential adsorption of VOCs onto porous materials. Furthermore, majority of studies use relatively high concentrations of VOCs in the gas phase, which typically results in capillary condensation and hysteresis. For instance, condensation and evaporation of isopropanol, heptane, and cyclohexane in mesoporous silica photonic crystals and the temporal response of the sensor were studied as a function of the heating rate and analyte concentration.^28^ This study measured the hysteresis of the optical response as the temperature of the sensor was cycled between 25 and 80 °C, which was characteristic to each analyte for partial pressures applied between 0 to 7.5 Torr (999.915 Pa). The capillary condensation of vapors was also studied in nanoporous alumina films with non-interconnected cylindrical pores open at one end by the same group.^29^ Those studies normally use high concentrations in the gas phase. However, for some applications, such as sensing, it is important to understand sorption properties and kinetics at lower concentrations than those previously reported.

In addition, the sequential adsorption of one VOC inside a nanoporous material immediately followed by another VOC has not been studied previously. This is important not just for improving our scientific understanding but also for real-world applications where the VOCs in the environment can change. Thus, it is imperative to understand whether this will result in a displacement of initially adsorbed compounds with the newly introduced compounds.

We report the study of real-time in situ adsorption and desorption kinetics of polar and nonpolar VOCs in mesoporous silica using optical reflectance spectroscopy. Isopropanol and nonane were chosen as polar and nonpolar VOCs to show how the compounds’ polarity and affinity to the silica surface play a role in sorption kinetics. We show adsorption–desorption kinetics for VOC concentrations of 1100 ppm for isopropanol and 630 ppm nonane. Those concentrations are lower than those that are commonly reported for VOC adsorption studies. The effect of mesoporous silica temperature was investigated in a temperature range between 20 and 70 °C, and sorption curves as a function of temperature were obtained. Sorption hysteresis was not observed, which is advantageous for sensing applications. Sequential adsorption studies of isopropanol followed by nonane and then nonane followed by isopropanol were measured in order to determine whether one compound will displace the other from the pores. The sequential introduction of VOCs provides information about the characteristics of silica pore occupancy when exposed to a polar and a non-polar molecule.

## 2 Experimental Methods and Sample Characterization

### 2.1 Optical Reflectance Measurements

**Figure 1:**
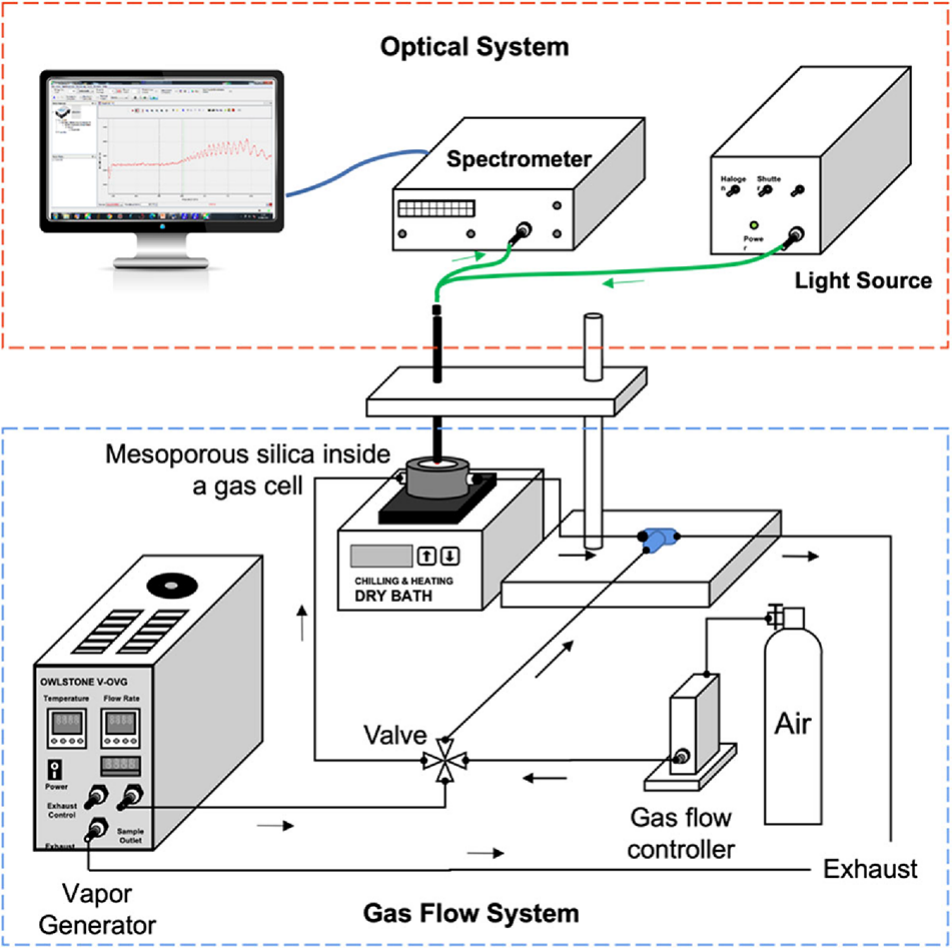
Schematic diagram of the experimental setup for the in situ sorption study.

The in situ sorption of VOCs inside mesoporous silica was measured using spectroscopic reflectance measurements. A tungsten halogen light source and Ocean Optics spectrometer (UV–VIS USB 2000) with a reflectance fiber-optic probe were used. The measured wavelength range was from 450 to 900 nm. A schematic illustration of the setup is shown in Figure 1. The reflectance spectrum exhibits an optical interference pattern (Fabry–Pérot fringes) as a function of wavelength arising from constructive and destructive interferences of light reflected from the top and bottom interfaces of the porous layer. The frequency of the periodic oscillation is proportional to the optical thickness (OT) of the porous layer, which is twice the product of the refractive index (*n*) and the thickness (*L*) of the layer on top of silicon. *n* of the porous layer corresponds to the effective refractive index of the porous layer that accounts for both the silica and the medium that fills the pores, according to Bruggeman effective medium approximation.^30,31^ Fourier transform was applied on the reflectance spectrum as a function of frequency, which results in a single peak at a position of 2*nL*, which is the OT of the layer. A spectral shift of the 2*nL* peak position occurs when the refractive index of the porous silica layer changes, which is when air in the pores is replaced by volatile organic compounds.^30,32,33^ A Python script was written for analysis of the peak position over time and for plotting the results.

### 2.2 Gas Setup

The porous silica sample was placed inside an air-tight metallic gas cell with an optical quartz window on the top for optical reflection measurement. The temperature of the mesoporous silica was controlled using a cold/hot dry bath. Before each experiment, the sample was cleaned by heating in an oven to 300 °C for 6 h. The vapor flowed via a Teflon tubing horizontally into a metallic gas cell positioned on the heating/cooling dry bath. A vapor generator (V-OVG, Owlstone) (temperature range: 25–100 °C) was used to generate and regulate the concentration of vapors in the parts-per-million (ppm) concentration. The four-way valve was used to switch between dry air and air with VOCs. A flow rate of 100 mL/min was used for all the experiments.

### 2.3 Fabrication of Mesoporous Silica

A boron-doped p-type silicon wafer with a resistivity of 0.01 Ohm-cm and crystallographic orientation of (100) was electrochemically etched in a 3:7 (*v*/*v*) mixture of 48% hydrofluoric acid and ethanol under a current density of 89.97 mA/cm^2^ for 285.71 s. Ethanol and pentane were used for cleaning the sample immediately after fabrication. The sample was then thermally oxidized in ambient air at 900 °C for 6 h, resulting in a porous silica layer on top of the silicon wafer.^3^

### 2.4 Mesoporous Silica Characterization

**Figure 2:**
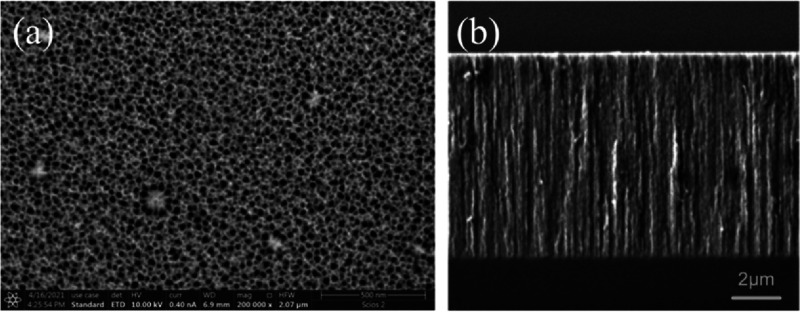
Scanning electron microscope (SEM) images of porous silica: (a) top-down view of the porous surface and (b) cross-sectional view of the 8 μm-thick mesoporous silica layer (middle) between silicon (bottom) and air (top).

**Figure 3:**
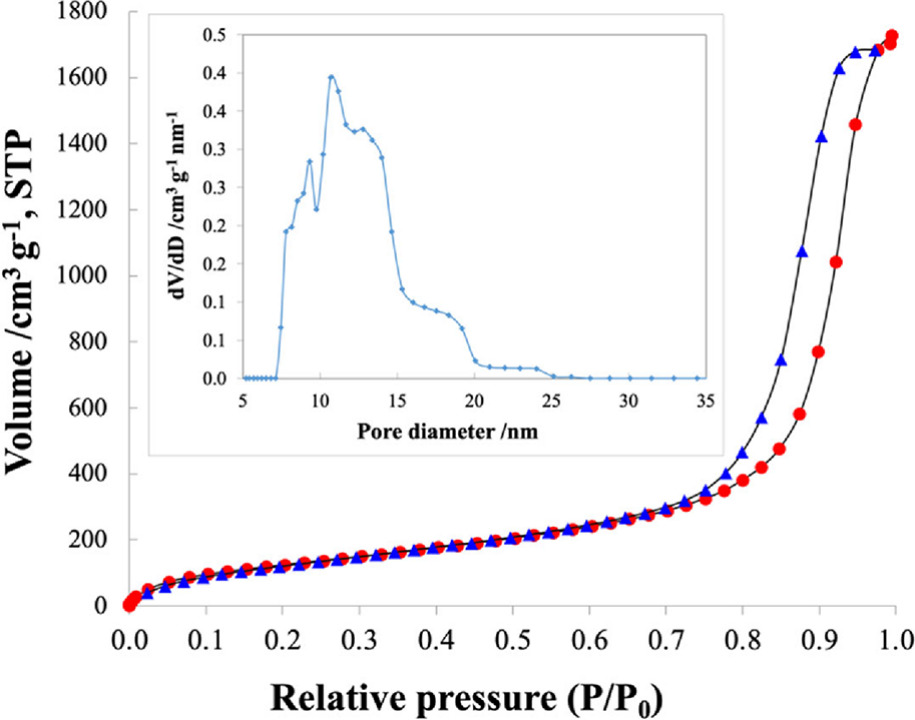
BET nitrogen adsorption–desorption isotherm plot for mesoporous silica. Inset: pore size distribution for mesoporous silica.

The top surface view and thickness of the mesoporous silica layer were observed by scanning electron microscopy (SEM). A dual focused ion beam/scanning electron microscopy system (Scios 2 HiVac system from Fisher Scientific) was used for high-resolution imaging of the upper surface of the sample as shown in Figure 2a. For a cross-section measurement, the sample was cleaved and gold-sputtered, vertically mounted on an SEM sample holder, and imaged using a Tescan VEGA3 SEM instrument. The image of the cross-section is shown in Figure 2b, where the 8 μm-thick porous layer is sandwiched between silicon and air. Nitrogen adsorption–desorption profiles were recorded at 77 K using a Quantachrome Autosorb IQ analyzer. The hysteresis pattern of the silica sample, shown in Figure 3, is similar to type IV hysteresis (IUPAC). The mesoporous silica surface area was found to be approximately 493 m^2^/g. The pore-diameter distribution curve shows that the pore diameters range between 7 and 20 nm with a dominant pore diameter of approximately 11 nm.

## 3 Results and Discussion

### 3.1 Effect of Mesoporous Silica Temperature on Gaseous Isopropanol and Nonane Sorption

The effect of mesoporous silica’s temperature on isopropanol and nonane sorption was studied at seven different temperatures between 10 and 70 °C. The measurements at each temperature were repeated three times. Isopropanol and nonane were selected for this study for their different polarities. The porous silica contains Si–OH and Si–OSi bonds that can interact with polar molecules via dipole–dipole interactions and hydrogen bonds, whereas non-polar molecules will only have a weak interaction with the silica surface.

The experiments were carried out with 1100 ppm isopropanol and 630 ppm nonane. Similar gas concentrations for both isopropanol and nonane could not be generated due to the differences in their vapor pressures and the limitations of the gas setup. The molecular mass, refractive index, vapor pressure, and concentration of isopropanol and nonane are shown in Table 1.

**Table 1:** Molecular Weight, Refractive Index, Vapor Pressure, and Concentration of Isopropanol and Nonane

VOC	molecular mass (g/mol)	refractive index at 25 °C	boiling point (°C)	vapor pressure (mmHg) at 25 °C	conc. (ppm)
isopropanol	60.1	1.3833^34^	82.5	45.4^35^	1100
nonane	128.2	1.4035^36^	151	4.3^35^	630

**Figure 4:**
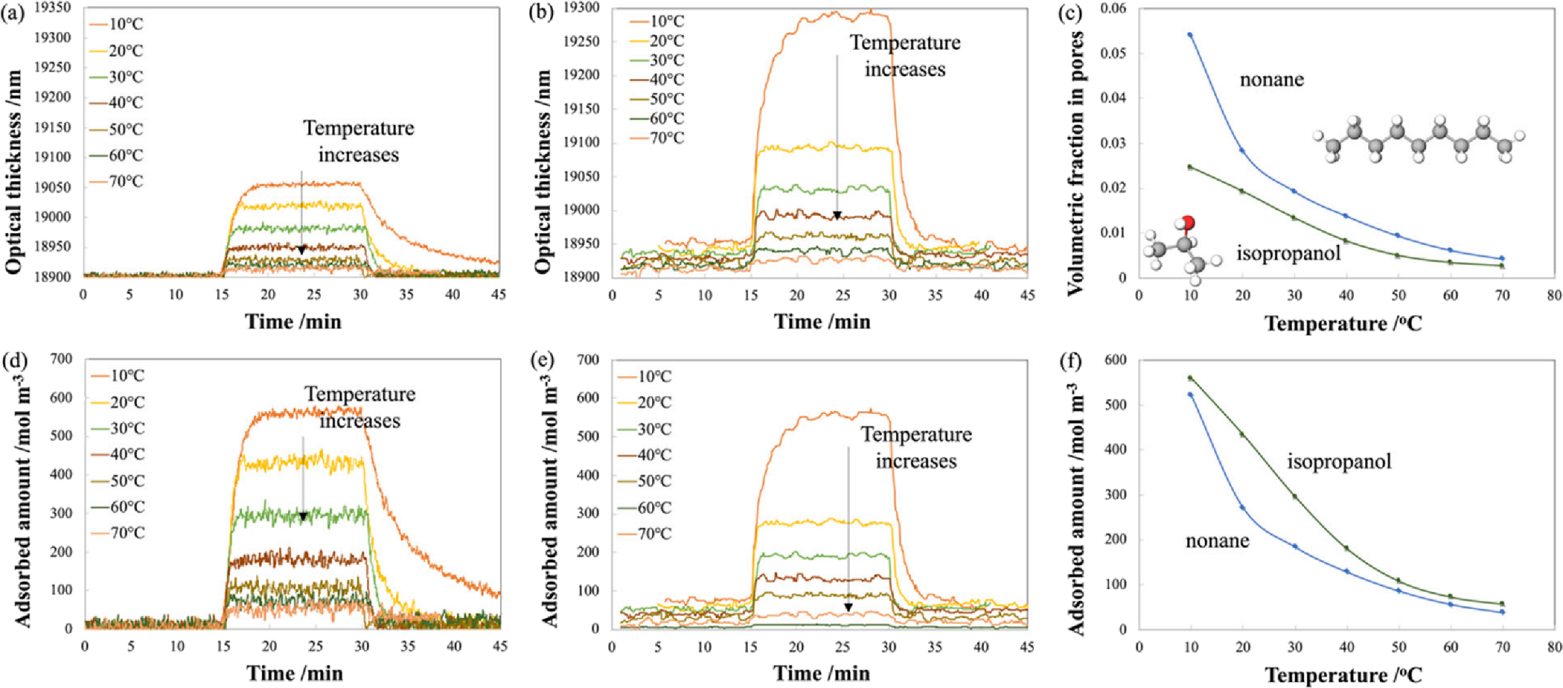
(a) Optical thickness as a function of time for 1100 ppm isopropanol in dry air for mesoporous silica temperatures between 10 and 70 °C. (b) Optical thickness as a function of time for 600 ppm nonane in dry air for mesoporous silica temperatures between 10 and 70 °C. (c) Volumetric fraction of VOCs in the pores at the steady state as a function of mesoporous silica temperature for 1100 ppm isopropanol (green curve) and 630 ppm nonane (blue curve) in dry air. (d) Calculated adsorbed amount of molecules as a function of time for 1100 ppm isopropanol in dry air for mesoporous silica temperatures between 10 and 70 °C. (e) Calculated adsorbed amount of molecules as a function of time for 600 ppm nonane in dry air for mesoporous silica temperatures between 10 and 70 °C. (f) Calculated adsorbed amount of molecules at the steady state as a function of mesoporous silica temperature for 1100 ppm isopropanol (green curve) and 630 ppm nonane (blue curve) in dry air.

Optical thickness (OT) curves of the porous layer as a function of time for isopropanol (at a concentration of 1100 ppm) are shown in Figure 4a, and for nonane (at a concentration of 630 ppm) in Figure 4b. In the aforementioned experiments, the porous silica sample was initially exposed to dry air then to VOC (at *time* = 15 min) and then back to dry air (at *time* = 30 min). For both VOCs, the OT increases when the VOCs were introduced into the gas cell, rapidly filling the pores and then reaching a steady-state condition. The steady-state OT value decreases as the temperature of the porous silica increases. Additionally, it can be seen that after switching to dry air (at time = 30 min), the desorption of isopropanol from the silica pores is slower compared to desorption of nonane, especially at lower temperatures. This can be attributed to the polar nature of isopropanol that has a much stronger interaction with the silica surface compared to nonpolar nonane. The porous SiO_2_ surface mainly comprises Si–O–Si and Si–OH bonds, which take part in hydrogen bonding with the adsorbed VOCs.

Overall, the measured OT values for nonane are higher than for isopropanol, even though nonane’s concentration in the gas phase is lower. The OT depends both on the number of adsorbed molecules and the refractive index of the adsorbed molecules, and the refractive index of nonane is higher than the refractive index of isopropanol. Bruggeman effective medium theory can be used for estimating the average or effective refractive index of a porous layer.^3,37,38^ The OT of porous silica exposed to dry air is ∼18.9 μm, and therefore, 2 · *n*_porous layer_ · *L* = 18.9 μm, while *L* = 8 μm (from SEM), yielding *n*_porous layer_ = 1.18. Now, a two-component Bruggeman model can be used to calculate the porosity of the porous silica layer
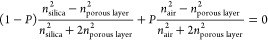
where *n*_air_ = 1 and *n*_silica_ = 1.458, resulting in porosity *P* = 0.5875 (i.e., 58.75%).

In order to calculate the volume fraction of the VOCs inside the pores (*V*) at each temperature, the three-component Bruggeman model can be used
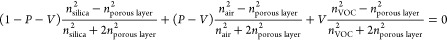
where *n*_isopropanol_ and *n*_nonane_ were taken at different temperatures from refs (39) and (40).

For the calculations, it was assumed that all VOC molecules inside the pores are adsorbed onto the pore walls and are not affecting the refractive index of the air, that is, *n*_air_ = 1 for the measured VOC concentrations. Practically, using this method, it is not possible to determine which portion of those molecules is adsorbed onto the silica surface or is present in the gas phase inside the pores. It should be noted that the OT will only be affected for VOCs adsorbed inside the pores rather than on top of the porous layer surface. VOCs adsorbed on top of the porous surface layer will not alter the effective refractive index of the porous media and thus will have no effect on the OT. The top surface area is also much smaller than the pore surface area, so, even if VOC molecules are adsorbed on the top surface layer, their relative number will be much smaller compared to molecules inside the pores.

The calculated volumetric fraction of VOCs in the pores (*V*), that is, the relative volume that the VOC molecules occupy in the pores, is plotted for isopropanol and nonane as a function of temperature in Figure 4c. For both VOCs, there is a decrease in *V* as the temperature increases, as expected. The small values of the volumetric fractions of VOCs inside the pores indicate that only a small number of molecules are adsorbed at all temperatures measured in this study. This can be attributed to the large surface area of the porous silica and the low concentrations of the molecules in the gas phase.

Because the pore diameters and volumes are much larger than the size of the molecules, any nanoconfinement effects can be neglected. Also, there is an equilibrium between the number of molecules in a gas phase and the number of molecules adsorbed in the pores for a given concentration and temperature. For our relatively low gas concentrations in the gas phase, it is expected that the number of adsorbed molecules will be small, which agrees with our results. It also should be noted that the measurement method used in this work is sensitive enough to be able to measure the adoption of even small number of molecules.

For the calculation of the number of molecules adsorbed, *N*, in moles per pore volume (mol/m^3^), the following formulas were used
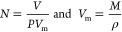
where *P* is the porosity, *V* and *V*_m_ are the volume fraction and molar volume of the adsorbed VOC, and *M* and ρ are the molar mass and the density of the adsorbed VOC. In the calculation, we use the liquid density of VOCs at atmospheric pressure at the same temperature as that of the silica skeleton.

The calculated adsorbed amount of molecules in moles per cubic meter of the pore volume as a function of time for 1100 ppm isopropanol in dry air for mesoporous silica temperatures between 10 and 70 °C is shown in Figure 4d. Similarly, the adsorbed amount of molecules as a function of time for 600 ppm nonane is shown in Figure 4e. The steady-state adsorbed amount of molecules as a function of mesoporous silica temperature is shown in Figure 4f. The number of adsorbed molecules of isopropanol is larger compared to the number of adsorbed nonane molecules as expected because the concentration of isopropanol is higher. In addition, isopropanol has a stronger affinity toward the silica surface, which can also contribute to the higher number of adsorbed isopropanol molecules. Both isopropanol and nonane molecules are much smaller in size compared to the average silica pore diameter (∼11 nm). Therefore, in this study, the molecular size or weight should not have a strong effect on the adsorption and desorption process.

### 3.2 Temperature Sorption Curves of Gaseous Isopropanol and Nonane in Mesoporous Silica

**Figure 5:**
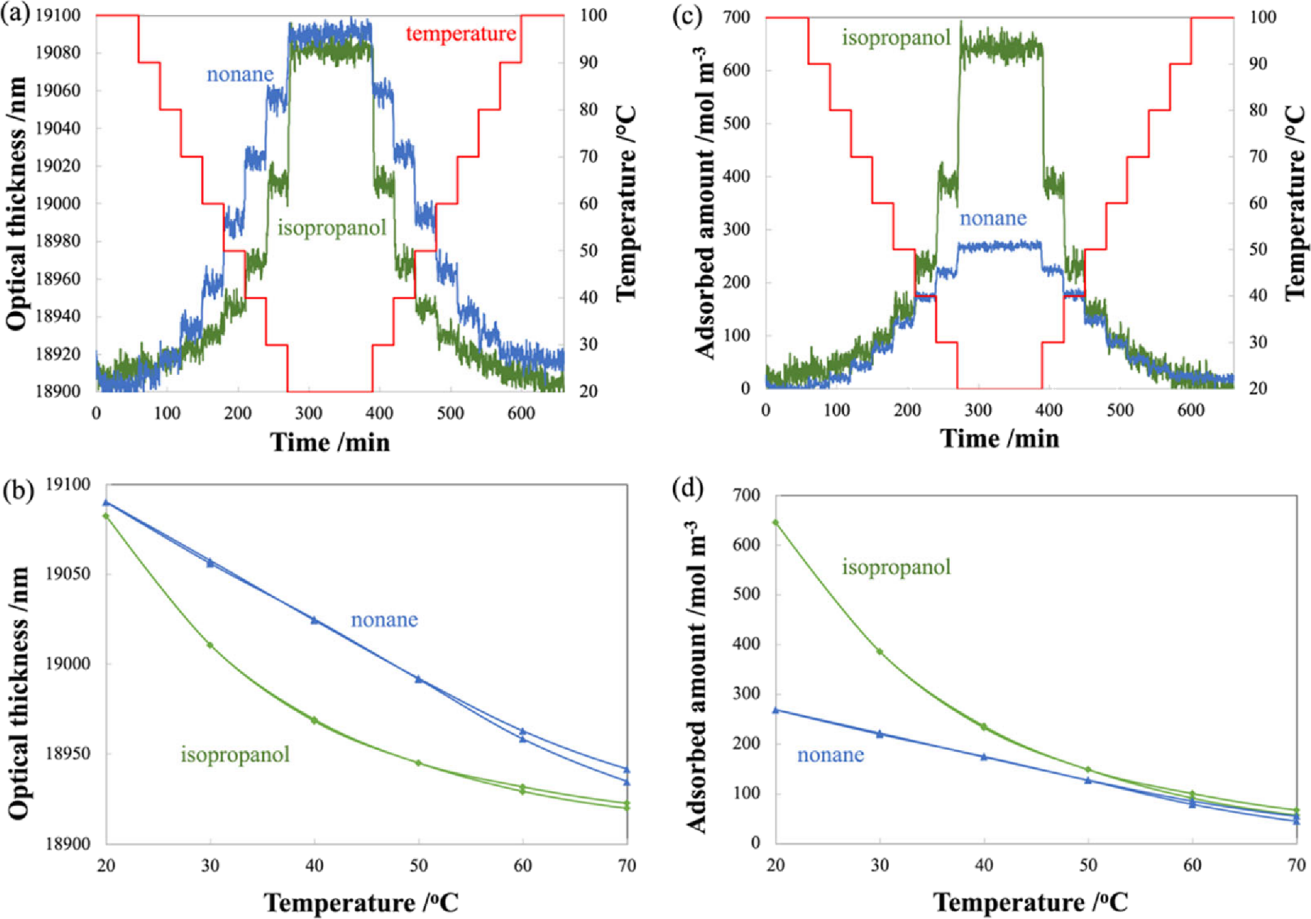
(a) Optical thickness and mesoporous silica temperature (red curve) as a function of time for isopropanol (green curve) and nonane (blue curve). (b) Steady-state optical thickness values for different mesoporous silica temperatures for isopropanol (green curve) and nonane (blue curve). (c) Calculated adsorbed amount of molecules and mesoporous silica temperature (red curve) as a function of time for isopropanol (green curve) and nonane (blue curve). (d) Steady-state adsorbed amount of molecules for different mesoporous silica temperatures for isopropanol (green curve) and nonane (blue curve). Concentrations of isopropanol and nonane were 1100 and 630 ppm, respectively.

The adsorption–desorption behavior as a function of the temperature of the mesoporous silica was studied at a constant gas flow and concentration of isopropanol or nonane. During the measurement, the temperature was gradually decreased from 100 down to 20 °C at 10 °C intervals and then gradually increased back. Figure 5a shows the temperature (red) of the porous silica and the measured OT curves for isopropanol (green curve) and nonane (blue curve). Each temperature step lasted 30 min to allow for a steady-state response. As expected, for both compounds, as the temperature decreases, the OT increases as more molecules adsorb in the pores, and similarly, as the temperature increases, the OT decreases. For temperatures higher than 70 °C, the noise levels are comparable to the change in OT due to temperature change (i.e., low signal-to-noise ratio), and thus OT values at 80, 90, and 100 °C were not considered for further discussion.

This experimental procedure was repeated three times for both VOCs, and the average steady-state OT values of the three repeats were calculated and are plotted in Figure 5b as a function of porous silica temperature. As the temperature of porous silica is decreased, more VOC molecules adsorb into the pores, and then upon increasing the temperature, VOC molecules desorb. The shapes of the curves are slightly different for isopropanol and nonane. The reason can be attributed to differences in polarity and their interaction with the polar silica surface. The nonpolar analyte nonane exhibits a close to linear trend between 20 and 70 °C, whereas isopropanol shows a more pronounced saddle-shaped curve. The discrepancies in the OT values at temperatures 60 and 70 °C can be attributed to the low signal-to-noise ratio.

The calculated adsorbed amount of molecules and mesoporous silica temperature (red curve) as a function of time for isopropanol (green curve) and nonane (blue curve) is shown in Figure 5c, and the steady-state adsorbed amount of molecules for different mesoporous silica temperatures for isopropanol (green curve) and nonane (blue curve) is shown in Figure 5d.

Hysteresis behavior as a function of temperature was not observed, which indicates that only a small number of molecules are adsorbed on the surface; thus, adsorption–desorption is only governed by the interaction of the molecules with the silica surface.

### 3.3 Sequential Adsorption of Gaseous Isopropanol and Nonane in Mesoporous Silica

**Figure 6:**
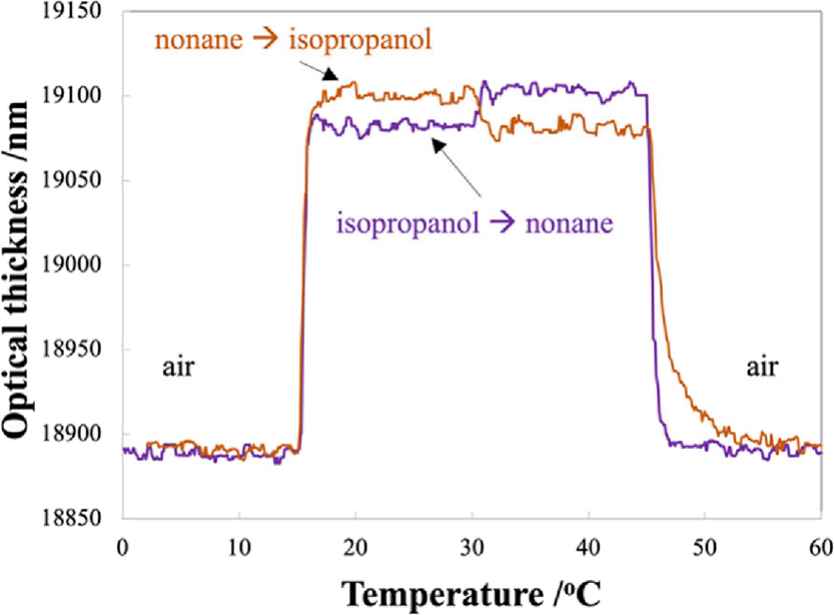
Optical thickness as a function of time for sequential sorption study for isopropanol followed by nonane (purple curve) and nonane followed by isopropanol (orange curve). The temperature of mesoporous silica was kept constant at 20 °C during the measurement. Concentrations of isopropanol and nonane were 1100 and 630 ppm, respectively.

In the previous sections, VOC sorption measurements were done on a clean mesoporous silica sample. In other words, pores of silica were empty, which enables VOCs to adsorb directly on the silica surface. Sequential sorption measurements were conducted to understand the effect of the presence of one VOC in the pore spaces on the subsequent adsorption of another. The temperature of the porous silica sample was kept constant at 20 °C throughout the measurement. Two sequential adsorption experiments were done, that is, one adsorption of isopropanol followed by nonane and the second adsorption of nonane followed by adsorption of isopropanol. The measured OT curves are shown in Figure 6, where the purple curve represents sorption of isopropanol first followed by nonane and the orange curve for nonane sorption followed by isopropanol. At time = 15 min, the first VOC is introduced into the gas cell. At time = 30 min, the first VOC was switched to another, and then at time = 45 min, VOC flow was changed to dry air. It can be seen that, in both cases, the steady state is reached within a few minutes. It can also be seen that the optical thickness in the presence of a certain VOC only depends on the properties of that VOC and is not affected by the previously adsorbed VOC. This indicates that the VOC that was introduced second has completely replaced the first VOC that was in the pores initially. At time = 45 min, when the VOC is switched to dry air, desorption occurs. The desorption profiles are different—nonane desorbs more rapidly compared to isopropanol, corresponding to previous results in Figure 4.

**Figure 7:**
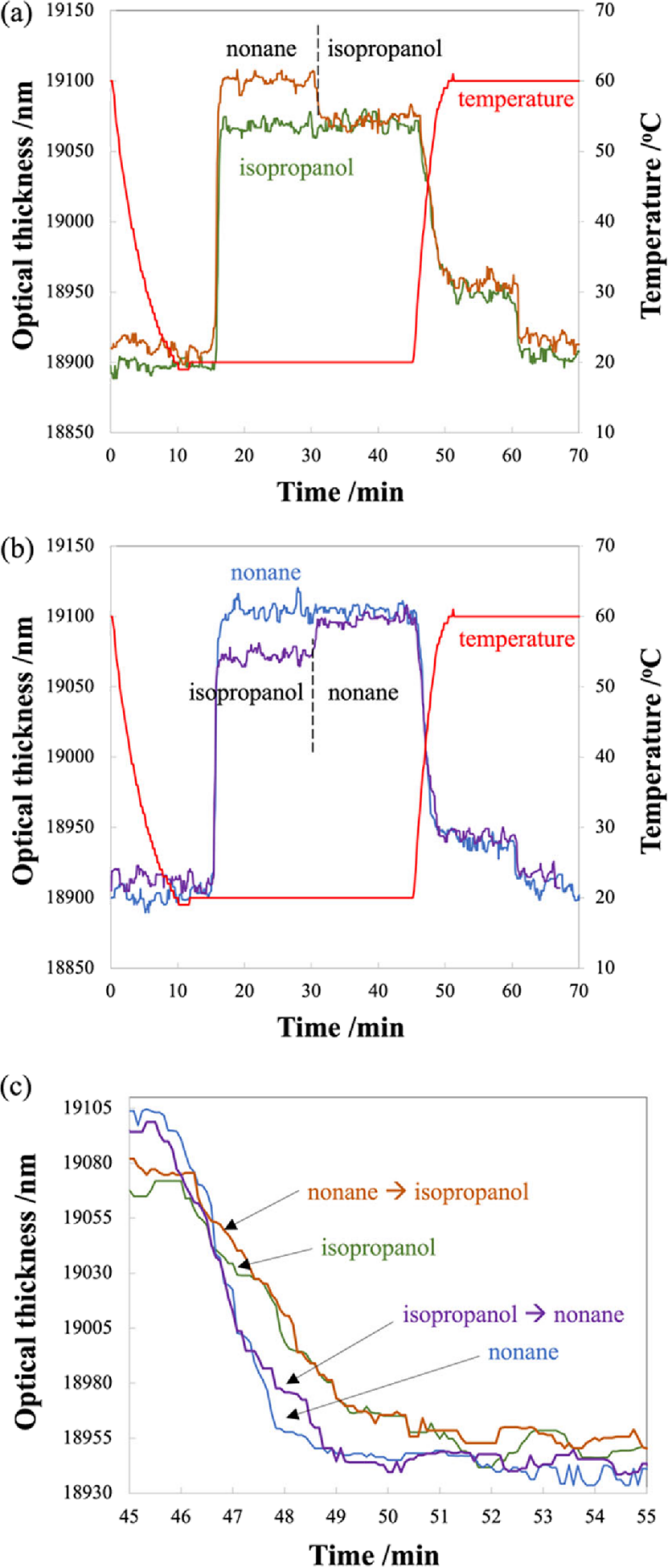
(a) Optical thickness and mesoporous silica temperature as a function of time for sequential sorption study for isopropanol (green curve) and nonane followed by isopropanol (orange curve). (b) Optical thickness and mesoporous silica temperature as a function of time for sequential sorption study for nonane (blue curve) and isopropanol followed by nonane (purple curve). The temperature of mesoporous silica was kept constant at 20 °C for 45 min and then increased to 60 °C. At 60 min, dry air was introduced. (c) Optical thickness during the temperature increase from 20 to 60 °C. Concentrations of isopropanol and nonane were 1100 and 630 ppm, respectively.

The measurement of sequential sorption was repeated with the difference that, this time, the temperature of porous silica was increased from 20 to 60 °C to facilitate desorption instead of switching to dry air. The four curves of OT as a function of time are shown in Figure 7 for isopropanol (green curve), nonane followed by isopropanol (orange curve), nonane (blue curve), and isopropanol followed by nonane (purple curve).

The results of the first 45 min agree with those plotted in Figure 6. At time = 45 min, the temperature of mesoporous silica was increased to 60 °C for 15 min, and desorption of VOCs occurred (Figure 7c). At time = 60 min, dry air was introduced, resulting in complete desorption and the OT returned to the initial value (i.e., no VOCs inside the pores). The desorption profiles between 45 and 60 min of nonane and nonane added following isopropanol sequentially are almost the same. Likewise, desorption profiles of isopropanol and isopropanol following nonane sequentially are almost the same. This further supports the statement that a complete replacement of molecules occurs when VOCs are switched. Overall, it can be said that, in these experimental conditions, despite the large difference of the VOCs’ polarities and VOCs’ interaction with silica surface, the second VOC molecules replace the first VOC. Such a behavior could be anticipated since the desorption of VOCs from mesoporous silica is quite rapid (within approximately 5 min upon switching from VOC to dry air). Thus, generally, the adsorption of VOCs is not very strong, facilitating rapid replacement of one VOCs over the other.

There is an equilibrium between the number of molecules in the adsorbed state and in the headspace. This equilibrium represents a state in which the rate of adsorption of molecules onto the pores is counterbalanced by the rate of desorption of molecules back into the gas phase. When we introduce a new VOC during sequential adsorption, the old VOC simply desorbs at its own rate. The two VOC molecules do not interact or compete with each other since the surface area is very high and the concentrations of VOC molecules are very low. For very high VOC concentrations, the molecules will most likely interact with each other in addition to the pore walls, and different behaviors could be observed.

The polarity of the VOC molecules affects the desorption curve regardless of whether it was followed by dry air or another VOC. As seen in Figure 7c, nonane has a more rapid desorption curve than isopropanol since isopropanol has a stronger interaction with the silica surface. From our experiment, the polarity of the VOC does not affect the molecular replacement as seen by comparing sequential adsorption measurement and singular adsorption.

A different behavior might be observed for materials that have much stronger interactions with the adsorbed molecules, such as metal–organic frameworks (MOFs)^41,42^ and so on activated carbon.^43^ In those cases, the replacement might take much longer time or may not happen at all. Those considerations are important when designing materials for applications such as separation or sensing. For the sequential adsorption measurements, the amount of the adsorbed molecules cannot be calculated as was done for single VOC adsorption in Figures 4 and 5. This is because, for the calculations of the amount of molecules adsorbed, we use characteristic properties of the VOCs (such as the refractive index). Once the flow of the first VOC is stopped and the second VOC is introduced, the relative adsorption of each compound cannot be accurately determined. However, by comparing with the control measurements, it is possible to infer that replacement of molecules occurs when one VOC is replaced by the other.

## 4 Conclusions

In situ studies of gaseous VOCs isopropanol and nonane sorption inside mesoporous silica for different conditions and temperatures were performed using optical reflectance spectroscopy. This method provides a convenient and simple approach for in situ measurement of adsorption of VOCs in the porous silica layer. Kinetics of adsorption and desorption for different porous silica temperatures between 10 and 70 °C showed that more VOC molecules are adsorbed at lower temperatures. The desorption of VOCs following exposure to dry air is slower for isopropanol than for nonane, which can be attributed to the stronger interaction of polar and hydrogen-bond forming isopropanol with the polar surface of mesoporous silica. Sorption as a function of porous silica temperature was also obtained, showing different sorption shapes for isopropanol and nonane. Such curves could potentially identify the analytes based on the differences in diffusion and adsorption properties of each VOC within the mesoporous silica for different temperatures. Sequential sorption measurements with isopropanol and nonane indicate that the VOC that was introduced second has completely replaced the first VOC that was in the pores regardless of the strength of the interaction of the VOC with the silica surface.
